# Characterization and Discrimination of Ancient Grains: A Metabolomics Approach

**DOI:** 10.3390/ijms17081217

**Published:** 2016-07-27

**Authors:** Laura Righetti, Josep Rubert, Gianni Galaverna, Silvia Folloni, Roberto Ranieri, Milena Stranska-Zachariasova, Jana Hajslova, Chiara Dall’Asta

**Affiliations:** 1 Department of Food Science, University of Parma, Parco Area delle Scienze 95/A, 43124 Parma, Italy; laurarighetti@live.it (L.R.); gianni.galaverna@unipr.it (G.G.); 2 Department of Food Analysis and Nutrition, Faculty of Food and Biochemical Technology, University of Chemistry and Technology, Prague, Technicka 3, 166 28 Prague 6, Czech Republic; zacharim@vscht.cz; 3 Open Fields Srl, Strada Consortile 2, Collecchio, 43044 Parma, Italy; s.folloni@openfields.it (S.F.); info@openfields.it (R.R.)

**Keywords:** small grains, non-targeted metabolomics, phenolic lipid compounds, lipidomics, foodomics

## Abstract

Hulled, or ancient, wheats were the earliest domesticated wheats by mankind and the ancestors of current wheats. Their cultivation drastically decreased during the 1960s; however, the increasing demand for a healthy and equilibrated diet led to rediscovering these grains. Our aim was to use a non-targeted metabolomic approach to discriminate and characterize similarities and differences between ancient *Triticum* varieties. For this purpose, 77 hulled wheat samples from three different varieties were collected: Garfagnana *T. turgidum* var. *dicoccum* L. (emmer), ID331 *T. monococcum* L. (einkorn) and Rouquin *T. spelta* L. (spelt). The ultra high performance liquid chromatography coupled to high resolution tandem mass spectrometry (UHPLC-QTOF) metabolomics approach highlighted a pronounced sample clustering according to the wheat variety, with an excellent predictability (*Q*^2^), for all the models built. Fifteen metabolites were tentatively identified based on accurate masses, isotopic pattern, and product ion spectra. Among these, alkylresorcinols (ARs) were found to be significantly higher in spelt and emmer, showing different homologue composition. Furthermore, phosphatidylcholines (PC) and lysophosphatidylcholines (lysoPC) levels were higher in einkorn variety. The results obtained in this study confirmed the importance of ARs as markers to distinguish between *Triticum* species and revealed their values as cultivar markers, being not affected by the environmental influences.

## 1. Introduction

Cereals represent one of the most important commodities providing basic nutrients to human diet, such as corn, rice, sorghum, or wheat, whose starchy grains are used as food. Cereals are annual plants, and cereal crops must be reseeded for each growing season. These cereal grasses, domesticated during the Neolithic period, formed the basis of systematic agriculture. In the particular case of *Triticum* species, they have been classified into hulled and free-threshing (“naked”) forms. Among the latter, bread and durum wheat are the most important *Triticum* species cultivated worldwide [1].

On the one hand, “hulled wheats”, which means that the kernel retains its husk during harvest, were the earliest domesticated wheats by mankind and are the ancestors of current wheats. Ancient wheat cultivation drastically decreased during the 1960s due to dietary and economic changes, as well as the introduction of bread and durum wheat, which are both higher yielding [2]. However, during the past years, the increasing demand for natural and organic products led to the rediscovery of ancient wheat species such as spelt (*Triticum spelta* L.), emmer (*Triticum dicoccum* L.), and einkorn (*Triticum monococcum* L.) [3]. This renewed interest is associated with the desire for a healthy and equilibrated diet, such as the Mediterranean diet. In fact, hulled wheat has been recognized as a dietetic and healthy cereal, and it is recommended in treatment of disease related to high blood cholesterol, colitis, and allergies [3]. A comparison of ancient and standard wheat highlighted that the ancient grains are characterized by a higher content of soluble dietary fiber, proteins, and lipids (mostly unsaturated fatty acids) [4]. In addition, ancient wheats provide a much greater proportion of rapidly digestible starch (RDS) and higher starch digestion index (SDI) compared to bread wheat [5,6]. Concerning trace elements, emmer, einkorn, and spelt mainly differed from wheat cultivars for higher contents of Li, Mg, P, Se, and Zn [7].

Another additional benefit might be connected with a relatively high concentration of antioxidant compounds, which can contribute to the excellent nutritional properties of the hulled wheats. Among these phenolic compounds, alkylresorcinols (ARs) represent one of the major groups that are found in high levels in the outer layers of the kernels [8]. The impact of ARs have been studied for wholegrain wheat and rye, because these layers are mostly removed during flour production [9]. Furthermore, the C17:0/C21:0 ARs homologue ratio has been proposed to differentiate between common and durum wheats [8,9]. Recently, the concentration of saturated ARs allowed the differentiation of *Triticum* species according to their degrees of ploidy [10]. In particular, the levels of all ARs homologues significantly differed between hexaploid (bread wheat and spelt), tetraploid (durum and emmer), and diploid (einkorn) species.

Up to now, targeted methods, developed for quantification of a given class of metabolites, have been exclusively applied to investigate differences between ancient *Triticum* varieties [9,10]. Nevertheless, nowadays, advanced analytical tools have permitted the simultaneous analysis of hundreds of metabolites, allowing a better characterization of small molecules (up to 1200 Da), therefore, the composition of complex plant matrices can be investigated in-depth [11]. In fact, in the last decade, the applicability of metabolomics to food science and nutrition research has strongly emerged [11,12,13,14,15,16].

In the present study, a metabolomic untargeted method was developed to investigate a broad spectrum of ancient wheats compounds in order to determine the relative roles of genotype and environment in determining the metabolites composition. Identifying similarities and differences that permit to distinguish between ancient *Triticum* varieties may be useful for the determination of nutritional aspects and adulterations, since emmer and einkorn are more expensive than spelt. For this purpose, 77 hulled wheat samples were analyzed using a non-targeted metabolomics approach based on solid liquid extraction followed by a reversed phase liquid chromatography separation coupled to quadrupole-time-of-flight mass spectrometer (LC-QTOF), and multivariate data analysis.

## 2. Results

### 2.1. Multivariate Modeling

**Figure 1:**
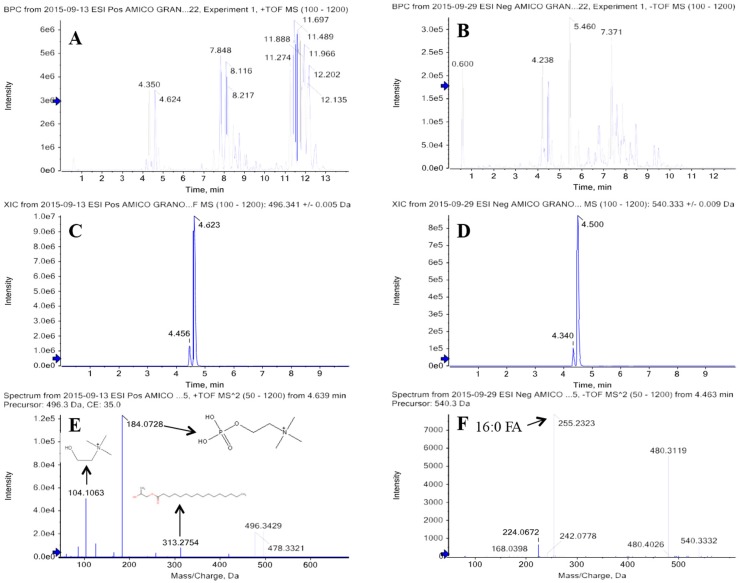
Ultra high performance liquid chromatography coupled to high resolution tandem mass spectrometry base peak chromatograms of ancient wheat extract obtained using positive (**A**) and negative (**B**) ionization modes. Extracted ion chromatogram (XIC) of Lyso PC 16:0 ionized in positive ([M + H]^+^ *m*/*z* 496.3399) (**C**) and negative ([M + HCOO]^−^ *m*/*z* 540.3332) (**D**) modes. Product ions acquired automatically by the information-dependent acquisition (IDA) method for the *m*/*z* 496.3399 (**E**) and *m*/*z* 540.3332 (**F**) parent ions. Blue arrows are thresholds and indicators in terms of RT and *m*/*z* values.

To perform sample classification, at first all 77 chromatograms were independently aligned for both polarities (see Figure 1). This returned a primary dataset with 4191 and 3253 features for positive and negative modes, respectively. Afterward, data reduction was performed based on previous work [12]. The primary filtering step excluded the background peaks present in blank samples. Then, in order to remove signal redundancy, only monoisotopic peaks were considered. The third filtering step was performed by choosing all the molecular features present in at least 50% of the samples in one group. This last step removed 2051 peaks for positive mode and 1666 for negative mode, representing approximately 50% of the original dataset, leaving 686 and 490 peaks for positive and negative, respectively.

**Figure 2:**
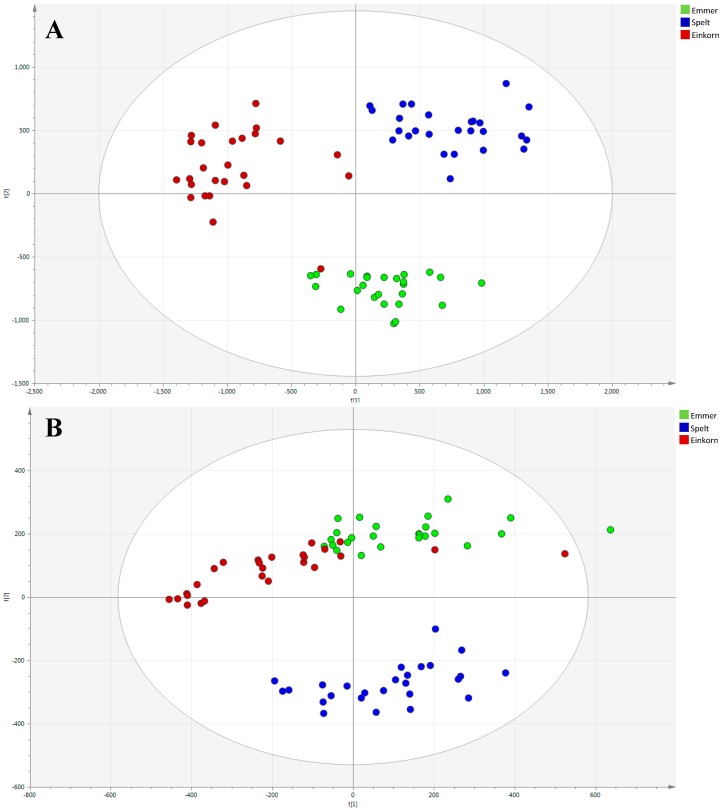
Unsupervised principal components analysis (PCA) models built from positive (**A**) and negative (**B**) ionization data set. Red dots: Einkorn (ID331). Green dots: Emmer (Garfagnana). Blue dots: Spelt (Rouquin).

At this point, the principal components analysis (PCA) models were built to investigate the metabolome, and therefore, differences between all three classes of wheat. The mechanism, already explained elsewhere [13], is based on the ability of the PC model to cluster samples in an unsupervised approach, since no information on group identity is used to construct the model. The PCA score plot obtained for positive and negative ionization modes are summarized in Figure 2. The first two principal components (PC) explained 50% of the total variance of the ESI(+) (32.9% and 17.1% for the PC1 and PC2, respectively) and 47.2% of the ESI(−) model (25.8% and 21.4% for the PC1 and PC2, respectively).

Samples were arranged in three major groups, indicating a sample clustering according to the varieties: emmer, einkorn, and spelt. A more pronounced clustering, among sample classes, was obtained in the ESI(+) data, as it can be seen in the PCA score plot (Figure 2A), even if one sample from ID331 is mixed up with Garfagnana variety. One out of 77 samples fell outside the 95% confidence ellipse, as it is shown in the ESI(−) PCA score plot (Figure 2B). This is considered a “moderate” outlier, while samples out of the confidence interval value of 99% (critical limit) are “strong” outliers. For this reason, this outlier was kept into the data set. No clustering according to vegetative year, growing location, and farming condition was found.

**Figure 3:**
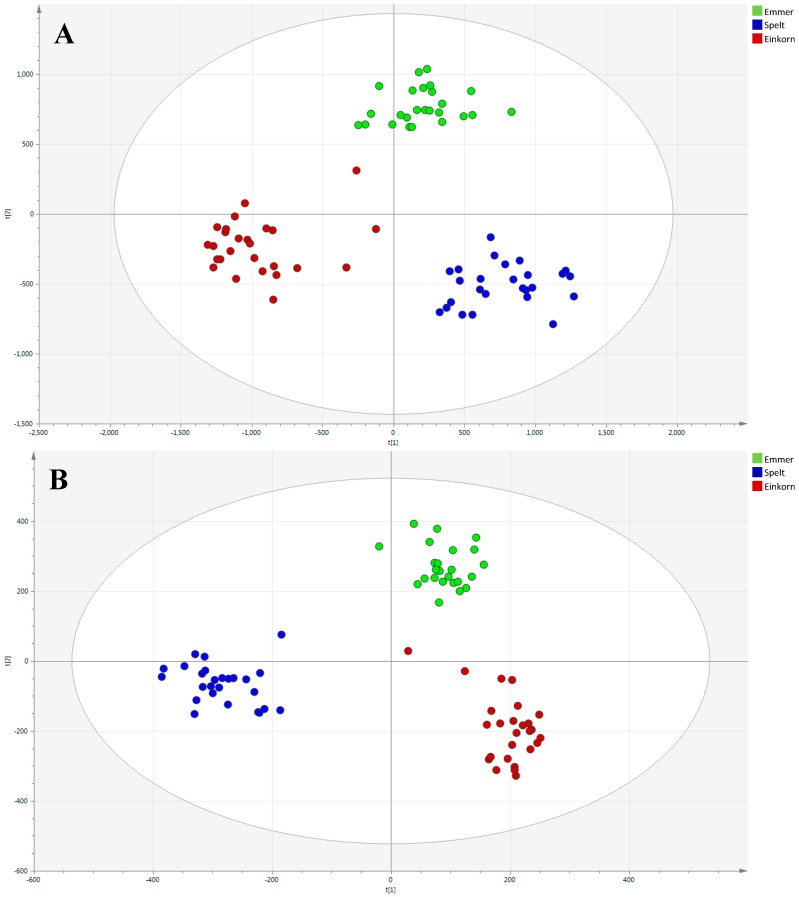
(**A**,**B**) PLS-DA model built with positive ionization data (*R*^2^*X* = 0.578, *R*^2^*Y* = 0.942, *Q*^2^ = 0.916) and negative ionization data (*R*^2^*X* = 0.709, *R*^2^*Y* = 0.967, *Q*^2^ = 0.944). In both ionization modes these three varieties were clearly separated. Red dots: Einkorn (ID331). Green dots: Emmer (Garfagnana). Blue dots: Spelt (Rouquin).

The differences between these three varieties were confirmed when partial least squares discriminant analysis (PLS-DA) (see Figure 3) and orthogonal partial least squares discriminant analysis (OPLS-DA) models were constructed. PLS-DA was performed to maximize differences and OPLS to highlight key variables and potential biomarkers. The quality of the models was excellent as shown in Table 1, where all the goodness of fit (*R*^2^) and the prediction ability (*Q*^2^) parameters are summarized. PLS-DA models highlighted highly quality parameters that were not significantly improved to OPLS-DA models, suggesting a low “structure noise” in the data set. OPLS-DA has the capacity to improve prediction ability because it separates out the structured noise, which is modeled separately.

The high *Q*^2^ values obtained for both supervised models indicated excellent predictabilities and suggested that the metabolomics approach applied was able to reveal differences between the grain varieties studied.

Moreover, in order to avoid the risk of overfitting, each generated model was validated by cross-validation tool [14], using the leave 1/3 out approach. Misclassification tables (see Table S1) indicate that 100% of ancient wheat lines (three out of three) were correctly classified in the ESI(–) data, while in ESI(+) OPLS-DA model the percentage of total correct classification was 98.7%, as one sample was not correctly predicted. 

### 2.2. Discriminant Metabolites Identification

In order to obtain relevant information regarding the metabolic differences between the varieties, a limited set of statistically meaningful metabolites had to be selected. In the present study, discriminant markers selection was performed merging the metabolites resulting from the PLS-DA loadings plot with those obtained using the Variable Influence in Projection (VIP threshold > 1.5). The identity of compounds that were found to be significant in class separation was confirmed by ultra high performance liquid chromatography coupled to high resolution tandem mass spectrometry (UHPLC-HRMS) analysis based on accurate MS and MS/MS data, as well as theoretical and experimental isotopic patterns were evaluated in-depth. Features were searched against the METLIN, KEGG, LIPIDMAPS and HMDB online databases [11]. At the same time, empiric formulae of the unknown compounds were calculated by Formula Finder option in Peak View software (version 2.2, SCIEX, Concord, ON, Canada) aiding to confirm or refuse potential structures. Subsequently, comparison of the fragmentation pathway of the proposed compound, found in the above-mentioned databases, with the fragments experimentally obtained confirmed the identity.

All metabolites identified are summarized in Table 2 including tentative identification, pseudomolecolar ion, retention time, mass error (ppm), higher metabolite intensities associated with ancient grain varieties, and VIP values. For all metabolites identified calculated mass error (Δppm) was lower than 4 ppm.

In the present work, seven statistically significant markers, belonging to the resorcinol’s class, were tentatively identified. The seven ARs were detected in negative ionization mode producing both a [M − H]¯ and the [M + HCOO]¯. Since these metabolites are commonly detected by GC-MS [17] or HPLC-UV [18] techniques, MS/MS spectra were not available in the online database. Thus, we tentatively identified them checking the exact mass (mass error less than 1.7 ppm), the match of experimental and theoretical isotope pattern in terms of spacing and relative intensities, and the most abundant fragment ion [M − C_2_H_2_O]¯ yielded from the resorcinol ring, resulting from the neutral loss of 42 Da (see Figure S1) [19].

For the lipid identification, LipidView software (version 1.3 beta, SCIEX) was employed. Diacylglycerols (DGs) and triacylglycerols (TGs) were detected in positive mode as ammonium adducts, giving a pseudomolecular ion [M + NH_4_]^+^. Identification of 1-palmitoyl-2-linoleoyl glycerol was based on the accurate *m*/*z* 610.5405 [M + NH_4_]^+^, theoretical and experimental isotopic patterns and on the product ions *m*/*z* 337.2737 and *m*/*z* 313.2737 corresponding to the loss of palmitic and linoleic acid, respectively. The mass spectrum of a TG contained two different fatty acids; 1,2-dipalmitoyl-3-linoleoyl glycerol (*m*/*z* 848.7708), and two DG ions (*m*/*z* 551.5034). Similarly, the MS/MS spectrum of 1-palmitoyl-2-oleoyl-3-eicosenoyl-glycerol (*m*/*z* 904.8339), as it contains three different fatty acid species, exhibited three DG ions (*m*/*z* 631.5660, *m*/*z* 605.5503 and *m*/*z* 577.5190) [15,16].

Phospholipids were detected in both ionization modes and confirmed by ESI(+) with a characteristic fragment ion of *m*/*z* 184.0739 for phosphatidylcholines (PC) and *m*/*z* 184.0739, *m*/*z* 104.1078, *m*/*z* 86.0974 *m*/*z* for lysophosphatidylcholines (lysoPCs) [20]. Lyso PC (16:0) fragmentation pattern that allows identification of the compounds, is depicted in Figure 1.

## 3. Discussion

### 3.1. Phenolic Compounds

**Figure 4:**
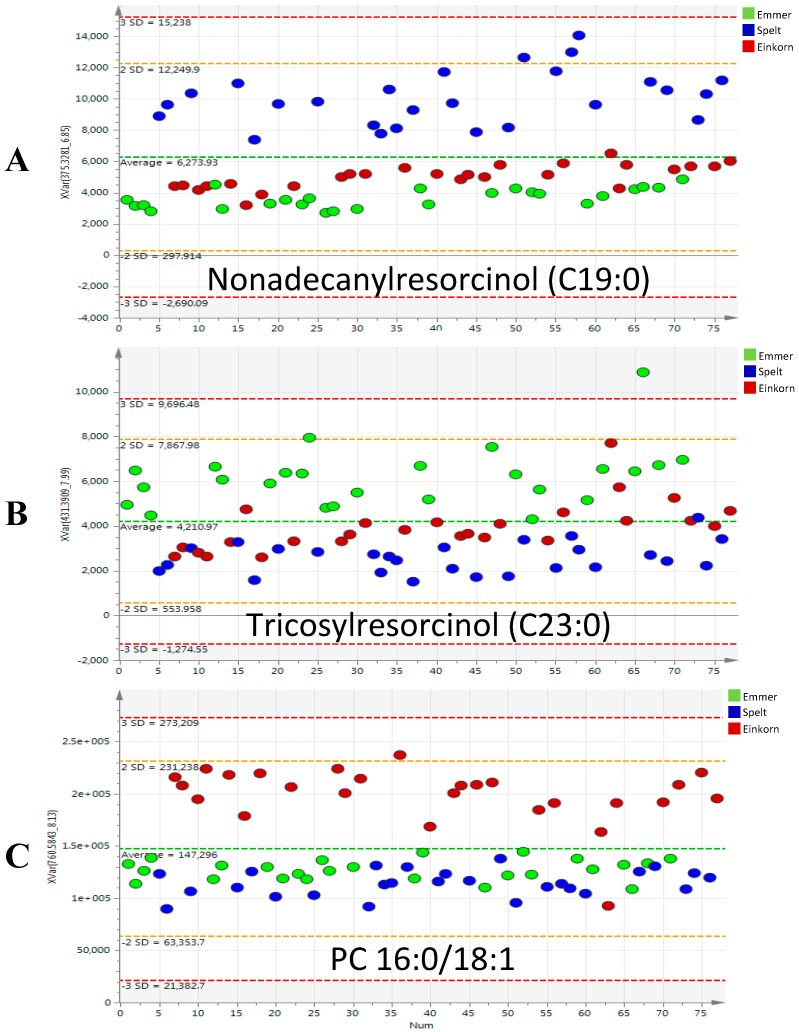
Variable trend plots of the most discriminant markers: nonadecanylresorcinol (C19:0), overexpressed in the spelt variety Spelt (Rouquin) (**A**), tricosylresorcinol (C23:0) marker having the highest influence to discriminate emmer variety (Garfagnana) (**B**) and PC (16:0/18:1), significantly higher in the einkorn variety (ID331) (**C**).

According to our results, ARs composition significantly differs between the studied varieties. In particular, two ARs, C21:0 and C19:0, turned out to be the most useful homologues to discriminate spelt from emmer and einkorn, as illustrated in the variable trend plot (Figure 4A). This is consistent with the results reported in the HEALTHGRAIN study [21], since, among the *Triticum* spp., spelt showed higher maximum values of ARs content ranging from 490 to 741 µg/g with C21:0 (approximately 47%) and C19:0 (36%) as the predominantly homologues found.

Spelt wheat, Rouquin, showed a distribution of AR homologues similar to that of common wheat, in agreement with earlier studies [21,22], being both hexaploid species [10]. By contrast, C23:0 (see Figure 4B) and C25:0 had the highest influence to discriminate emmer variety, Garfagnana, showing the same homologue pattern of durum wheat, characterized by the influence of the longer homologues. These longer AR homologues, which were isolated from a cereal bran-milling fraction, have been found efficient inhibitors of 3-phosphoglycerate dehydrogenase. Note that 3-phosphoglycerate dehydrogenase is a key enzyme of triglyceride synthesis, in adipocytes [23]. Also for this reason, the intake of ARs is considered beneficial as it reduces the absorption of cholesterol, regulate metabolism of triacylglycerols and affect levels of lipid-soluble vitamins [24].

ARs with modified alkyl chains are also present in cereals. These are believed to differ from ARs only in side-chain unsaturation or oxidation. On average, 15%–20% ARs contain unsaturated hydrocarbon chains as well as ketone and hydroxyl groups [21,22,23]. In the present study, two alk(en)yl resorcinols, nonadecenyl-resorcinol, and heneicosenyl-resorcinol, were identified and contributed to the clustering and differentiation of spelt, Rouquin. These AR analogues are suggested to be more bioactive than normal saturated ones [24].

ARs are found mainly in the outer layers (bran fraction) of cereal grains, which means that they are largely missing in refined cereal flour and conventional cereal products. Taking into account that these ancient grain varieties are mostly consumed in the form of whole grain, ARs may be present in food in high enough concentrations to have a bioactive effect.

ARs are absorbed in the small intestine of pig with an ileal recovery that varies between 21% and 40%, with no major difference between different chain-length homologues [25]. In fact, their metabolized forms have been found in human plasma and urine [26] suggesting that ARs might exert their biological effect in human after whole grain intake. 

Interestingly, our data suggests that sample clustering was not affected by growing location, organic or conventional farming and vegetative year, as it was previously reported [27]. Thus, the level of ARs metabolites was identified as a cultivar marker, strongly influenced by the genetic background, which is partially in line with Ziegler et al. [10]. In fact, they reported significant difference in the AR content of spelt grown in different location, whereas einkorn content did not differ among different location.

This outcome indicated a strong genetic influence on the AR homologue profile, suggesting that the metabolomics approach applied could potentially allow the determination of ancient wheat adulterations.

### 3.2. Glycerophospholipids and Glycerolipids

Among the statistically significant phospholipids, four molecular species (*m*/*z* 496.3399, *m*/*z* 520.3392, *m*/*z* 760.5851, *m*/*z* 758.5712) were found responsible for the separation of einkorn variety.

Two PCs, PC (16:0/18:2) and PC (16:0/18:1) were tentatively identified and trend plot of PC (16:0/18:1) is illustrated in Figure 4C. These results are consistent with a previous study [4], as einkorn was reported to show a richer lipid profiling among the ancient varieties, a lipid content 50% higher than those of bread wheat. In fact, PC 34:2 together with lysoPC 16:0 and lysoPC 18:2 are the major PC species detected, representing 60%–70% of the total wheat PC [28]. Acyl carbon and double-bond configurations in phospholipids are probably combination of the major fatty acids, that in einkorn are reported to be linoleic (18:2), oleic (18:1), and palmitic (16:0) acids [4]. In bread wheat, linoleic acid is the prevalent fatty acid too, however palmitic acid is more abundant than oleic acid. Consequently, einkorn lipids profile has a higher content of monounsaturated fatty acids (MUFA), lower content of polyunsaturated fatty acids (PUFA), and lower saturated fatty acids (SFA) that, from a nutritional point of view, contribute to the prevention of cardiovascular diseases, since MUFA and PUFA reduce thrombosis and atherosclerosis risk, influencing lipid and cholesterol synthesis [4].

## 4. Materials and Methods

### 4.1. Chemicals

The deionized water used for the LC mobile phase was obtained from a Milli-Q^®^ Integral system supplied by Merck (Darmstadt, Germany). High-performance LC (HPLC)-grade methanol, 2-propanol, dichloromethane, formic acid, and ammonium formate were supplied by Sigma-Aldrich (St. Louis, MO, USA).

### 4.2. Plant Material

For this study, three ancient wheat species have been chosen: Garfagnana *T. turgidum* var. *dicoccum* L. (emmer), ID331 *T. monococcum* L. (einkorn), and Rouquin *T. spelta* L. (spelt).

The most extensively cultivated species is *T. turgidum* ssp. *dicoccum*, which was largely grown in the hills and low mountain areas in Central and Southern Italy until the 19th Century, as reported by local tradition. The three varieties were cultivated in two locations in Emilia Romagna region, Parma and Bologna, in plots of 8.25 m^2^ with four replications. Grains were grown over two consecutive years (2013/2014 and 2014/2015) under two agricultural conditions: conventional (*n* = 23) and organic farming (*n* = 30) in Parma, whereas only conventional farming was applied in Bologna (*n* = 24). After harvesting, the whole grains were dried at ca. 10% humidity, stored at −20 °C and kept refrigerated until the analysis. Overall, 77 wheat samples were collected.

### 4.3. Metabolite Extraction

Wheat samples were ground into a fine powder using a ball mill (MM 301 Retsch, Haan, Germany). An amount of 1 g of ground wheat was weighed into a 50 mL polypropylene centrifugation tube, followed by the addition of 10 mL of a mixture of methanol/dichloromethane (50:50, *v*/*v*). After brief shaking, the content was stirred for 30 min at 240 strokes/min by a shaker (IKA Laborartechnik, Stufen, Germany). The tube was centrifuged (13,416 g) for 7 min (Rotina 35 R, Hettich Zentrifugen, Germany), then 1 mL of the extract was evaporated to dryness under a gentle stream of nitrogen. Finally, the residues were re-dissolved in 1 mL of isopropanol/methanol/water (60:30:5, *v*/*v*) prior to UHPLC-Q-TOF analysis. During the sample preparation blanks were also prepared for analysis consisting of all the steps mentioned above except for the addition of sample.

### 4.4. Quality Control (QC) Samples Preparation

In order to measure performance and system stability and assess the reproducibility of the sample treatment procedure, Quality Control samples (QC) were injected during the analyses.

QCs (*n* = 2) were obtained by mixing equal volumes (50 μL) of all 77 sample extracts and following the same procedure as for the other samples. QCs were injected at the beginning of the run and after every 10 real samples.

### 4.5. Ultra-High-Performance Liquid Chromatography-High Resolution Mass Spectrometry

UHPLC Dionex UltiMate 3000 RS system (Thermo Fisher Scientific, Waltham, MA, USA) coupled to a TripleTOF^®^ 5600 quadrupole time-of-flight (TOF) mass spectrometer (SCIEX) was employed for untargeted analysis of wheat. 

The chromatographic separation was performed using an Acquity BEH C18 column (Waters, Milford, MA, USA) 100 mm × 2.1-mm inner diameter, 1.7-μm particle size maintained at 60 °C. The mobile phases for metabolic analysis were the same for negative and positive electrospray ionization (ESI) modes. Gradient elution was performed by using 5 mM ammonium formate in Milli-Q water/methanol (95:5, *v*/*v*) (solvent A) and 5 mM ammonium formate in isopropanol/methanol/Milli-Q water (65:30:5, *v*/*v*) (solvent B) both acidified with 0.1% formic acid.

The following multistep elution gradient was used with both electrospray ionization (ESI) polarities: 0.0 min (10% solvent B; 0.40 mL·min^−1^) to 1.0 min (50% solvent B; 0.40 mL·min^−1^), subsequently 1–5 min (80% solvent B; 0.40 mL·min^−1^), 11.0 min, (100% solvent B; 0.50 mL·min^−1^). After a 4.5 min isocratic step, the system was re-equilibrated to initial conditions for 2.5 min (10% solvent B; 0.4 mL·min^−1^). The sample was permanently kept at 5 °C.

The ion source was a DuoSpray™ with a separated ESI ion source and APCI. ESI was used for the sample measurement and APCI was used for exact mass calibration of the TripleTOF instrument. In ESI(+) mode, the source parameters for metabolic analysis were as follows: capillary voltage, +4500 V; nebulizing gas pressure, 60 psi; drying gas pressure, 50 psi; temperature, 550 °C; and declustering potential, 80 V. The capillary voltage in ESI(−) mode was −4000 V, and the other source settings were the same as for ESI(+).

At the same time, a TOF MS method and information-dependent acquisition (IDA) method were used to collect MS and MS/MS spectra. The method consisted of a survey TOF MS experiment ranged from *m*/*z* 100 to 1200, in parallel, Product Ion (PI) spectra for the eight most intense ions of the survey spectra throughout the chromatographic run were recorded. Dynamic background subtraction was activated to acquire PI spectra of real eluted compounds, avoiding background ions. PI spectra were collected for ions ranging from *m*/*z* 50 to 1200. The PI spectra were recorded with collision energy of 35 V and collision energy spread of ±15 V was also set. In this way, both low-energy and high-energy fragment ions were present in a single spectrum. The total cycle time of the TOF MS and IDA methods was 0.55 s.

An automatic *m*/*z* calibration was performed by the calibration delivery system for every five samples using a positive or negative APCI calibration solution (SCIEX) according to the batch polarity. Each set of samples for each polarity was preceded by three blank controls: Milli-Q water, methanol and a blank (extraction procedure without the sample). Finally, the same MS approach was applied in ESI(−) mode. The resolving power achieved was greater than 31,000 (*m*/*z* 321.0192) full width at half maximum (FWHM) with both polarities. The PI spectra were measured in high-sensitivity mode, which provides half resolving power.

Instrument control and data acquisition were performed with Analyst 1.6 TF (SCIEX), and the qualitative analysis was performed using PeakView 2.2 (SCIEX) equipped with MasterView and Formula Finder and directly linked to the ChemSpider database, and LipidView software (version 1.3 beta, SCIEX) for lipid evaluation. The in-batch sequence of the samples was random (established on the basis of random number generation) to avoid any possible time-dependent changes during UHPLC-HRMS analysis, which would result in false clustering. To address overall process variability, metabolomics studies were augmented to include a set of eight sample technical replicates (10% of the samples set). Reproducibility analysis for compounds detected in these replicates provided a measure of variation for extraction, injection, retention time (RT), and mass accuracy.

### 4.6. Data Processing and Chemometrics Analysis

Data processing has been performed based on previous work [12]. Briefly, MarkerView software (version 1.2.1, SCIEX) was used for data processing (data mining, alignment, filtering, normalization, and Principal Component Analysis (PCA)) of the UHPLC-HRMS records. Data mining was performed based on an automated algorithm using RT window and peak finding; retention time (RT) range 0.4–13 min and *m*/*z* range 100–1200. In the next step, RT and *m*/*z* alignment of the respective peaks was carried out using RT and m/z tolerances of 0.2 min and 0.02 Da, respectively. Two separate positive and negative ionization data matrices, comprising lists of molecular features (called also peaks by MarkerView) characterized for each sample by (i) RT; (ii) *m*/*z* value; (iii) respective intensity and (iv) charge state (monoisotopic and isotopic), were automatically obtained using MarkerView, and subsequently total area sum normalization was performed for each sample. Prior to the actual PCA, data matrices were pre-processed using the Pareto scaling (the square root of the standard deviation is used as the scaling factor).

Orthogonal partial least squares discriminant analysis (OPLS-DA) employing the software SIMCA (v. 13.0, 2011, Umetrics, Umea, Sweden) was performed. The quality of the models was evaluated by the goodness-of-fit parameter (*R*_2_*X*), the proportion of the variance of the response variable that is explained by the model (*R*_2_*Y*) and the predictive ability parameter (*Q*_2_), which was calculated by a seven-round internal cross validation of the data using a default option of the SIMCA software. *R*_2_*X* and *R*_2_*Y* represent the fraction of the variance of *X* matrix and *Y* matrix, respectively, while *Q*_2_ suggests the predictive accuracy of the model. *R*_2_*X*, *R*_2_*Y*, and *Q*_2_ values close to 1 indicate an excellent model, and thus, from values higher than 0.5 indicate good quality of OPLS-DA models. In order to select the most significant and reliable variables, variable importance in the projection (VIP) was used. This parameter summarizes the importance of the *X*-variables, both for the *X*- and *Y*-models. In this research, VIP with the threshold >1.5 was used for selection of the most significant markers. VIP-values larger than 1 indicate important *X*-variables.

To avoid risk of overfitting, as the results found after Multivariate Data Analysis (MVDA) are sensitive to chance-correlations, statistical models have to be validated. For this reason, supervised models, OPLS-DA and PLS-DA, were validated by cross-validation, using the leave one-third out approach. The data set was divided into three parts and one-third of samples were excluded to build a model with the remaining two-thirds of samples. Excluded samples, one-third of samples, were then predicted by this new model and the procedure was repeated until all samples had been predicted at least once. Each time the percentage of correctly classified samples was calculated by generating a misclassification table.

## 5. Conclusions

In conclusion, differences in the metabolome of ancient grains were successfully detected using an untargeted UHPLC-HRMS metabolomics approach. Discriminant metabolites including alkylresorcinols, glycerophospholipids, and glycerolipids were identified allowing a metabolic characterization of ancient wheat grains.

The results obtained in this study confirmed the importance of different AR homologues as markers to distinguish between *Triticum* species. Furthermore, all the 15 identified molecules were revealed to be cultivar markers, strongly influenced by the genetic background, since their abundance was not significantly affected by growing location, organic or conventional farming, and vegetative year.

## Figures and Tables

**Table 1:** Statistical values for PCA, PLS-DA, OPLS-DA models. *R*^2^*X* (cum) and *R*^2^*Y* (cum) represent the variance of the *x* and *y* variables explained by the model, while *Q*^2^ is the cumulative predicted variation in the *Y* matrix.

Statistical Parameters	ESI(+) Models			ESI(−) Models		
	PCA	PLS-DA	OPLS-DA	PCA	PLS-DA	OPLS-DA
*R*^2^*X* (cum)	0.816	0.578	0.579	0.89	0.709	0.709
*R*^2^*Y* (cum)	-	0.942	0.942	-	0.967	0.967
*Q*^2^ (cum)	0.663	0.916	0.917	0.778	0.944	0.956

Principal components analysis (PCA), partial least squares discriminant analysis (PLS-DA) and orthogonal partial least squares discriminant analysis (OPLS-DA).

**Table 2:** Identification of discriminant metabolites between the three wheat varieties.

Biochemical Category	Biochemical Class	Tentative Identification	Pseudomolecolar Ion	*m*/*z*	RT (min)	Elemental Formula	Mass Error (Δppm)	Higher Metabolite Intensity in	VIP Value
Phenols	Resorcinols	Heptadecylresorcinol (C17:0)	[M − H]^−^	347.2956	6.3	C_23_H_40_O_2_	1.7	spelt	1.5
		Nonadecanylresorcinol (C19:0)	[M − H]^−^	375.3269	6.9	C_25_H_44_O_2_	1.5	spelt	4.2
		Nonadecenyl-resorcinol (C19:1)	[M − H]^−^	373.3112	6.3	C_25_H_42_O_2_	1.4	spelt	2.2
		Heneicosylresorcinol (C21:0)	[M − H]^−^	403.3582	7.4	C_27_H_48_O_2_	1.4	spelt	2.9
		Heneicosenyl-resorcinol (C21:1)	[M − H]^−^	401.3425	6.9	C_27_H_46_O_2_	1.3	spelt	1.5
		Tricosylresorcinol (C23:0)	[M − H]^−^	431.3895	8	C_29_H_52_O_2_	1.3	emmer	3.2
		Pentacosylresorcinol (C25:0)	[M − H]^−^	459.4208	8.5	C_31_H_56_O_2_	1.2	emmer	3.1
Glycerophospholipids (GLP)	Lysophosphatidylcholines (LysoPC)	LysoPC 16:0	[M + H]^+^	496.3399	4.5	C_24_H_50_NO_7_P	3.2	einkorn	4.3
		LysoPC 18:2	[M + H]^+^	520.3392	4.2	C_26_H_50_NO_7_P	1.2	einkorn	3.1
	Phosphatidylcholines (PC)	PC 16:0/18:1	[M + H]^+^	760.5851	8.2	C_42_H_82_O_8_NP	1.6	einkorn	2.9
		PC 16:0/18:2	[M + H]^+^	758.5712	7.9	C_42_H_80_NO_8_P	2.3	einkorn	3.9
	Phosphatidylinositols (PI)	PI 16:0/18:1	[M + H]+	835.5478	7.7	C_43_H_81_O_13_P	1.6	emmer	1.6
Glycerolipids (GL)	Diacylglycerols (DG)	DG 16:0/18:2	[M + NH_4_]^+^	610.5405	8.8	C_37_H_68_O_5_	1.5	emmer	4.9
	Triacylglycerols (TG)	TG 16:0/16:0/18:2	[M + NH_4_]^+^	848.7708	11.4	C_53_H_98_O_6_	1.8	spelt	3.1
		TG 16:0/18:1/20:1	[M + NH_4_]^+^	904.8339	12	C_57_H_106_O_6_	1.9	einkorn	3.8

Table columns: pseudomolecular ion = positive and negative ionization adduct; *m*/*z* = mass-to-charge ratio in daltons; RT = ion retention time in minutes; elemental formula = elemental composition of the neutral molecule; mass error ppm = Δ in ppm between the detected *m*/*z* and the theoretical *m*/*z*; higher metabolite intensity in = ion spectral intensity higher in emmer, einkorn, or spelt as indicated; VIP value = Variable Influence in Projection values.

## Supplementary Materials

Supplementary materials can be found at http://www.mdpi.com/1422-0067/17/8/1217/s1.

## Abbreviations

ARs	alkylresorcinols
QTOF	quadrupole time of flight
HRMS	high resolution mass spectrometer
QC	quality control
PCA	principal component analysis
PLS-DA	projection on latent structure-discriminant analysis
OPLS-DA	orthogonal projection on latent structure-discriminant analysis
DG	diacylglycerol
TG	triacylglycerol
PC	phosphatidylcholine
Lyso PC	lysophosphatidylcholine
