# Supplementary material for: Characterization and Discrimination of Ancient Grains: A Metabolomics Approach
Source: Int J Mol Sci. 2016 Jul 27;17(8):1217. doi: 10.3390/ijms17081217 (PMC5000615; doi:10.3390/ijms17081217)
Supplement: Supplementary file 1 [file ijms-17-01217-s001.pdf]

Supplementary Materials: Characterization and Discrimination of Ancient Grains: A Metabolomics Approach

Laura Righetti, Josep Rubert, Gianni Galaverna, Silvia Folloni, Roberto Ranieri, Stranska-Zachariasova, Jana Hajslova and Chiara Dall'Asta

Table S1. Misclassification tables related to OPLS-DA models. Panel A shows that one sample from the ID331 (einkorn) was mixed up with the group of Garfagnana (emmer), decreasing the percentage of total correct classification up to 98.7 in the ESI(+) OPLS-DA model. Panel B indicates that 100% of ancient wheat lines (3 out of 3) were correctly classified in the ESI(−) data. Low probabilities ($p = 4.30 \times 10^{-27}$ and $p = 7.10 \times 10^{-27}$) of random table generation, assessed by Fisher's Exact Probability, were found for both ESI models.

A	Members	Correct	Garfagnana	Rouquin	ID331
Garfagnana	25	100%	25	0	0
Rouquin	26	100%	0	26	0
ID331	26	96.15%	1	0	25
No class	0		0	0	0
Total	77	98.70%	26	26	25
Fishers prob.	4.30×10^{-27}				
**B**	**Members**	**Correct**	**Garfagnana**	**Rouquin**	**ID331**
Garfagnana	25	100%	25	0	0
Rouquin	26	100%	0	26	0
ID331	26	100%	0	0	26
No class	0		0	0	0
Total	77	100%	25	26	26
Fishers prob.	7.10×10^{-27}				

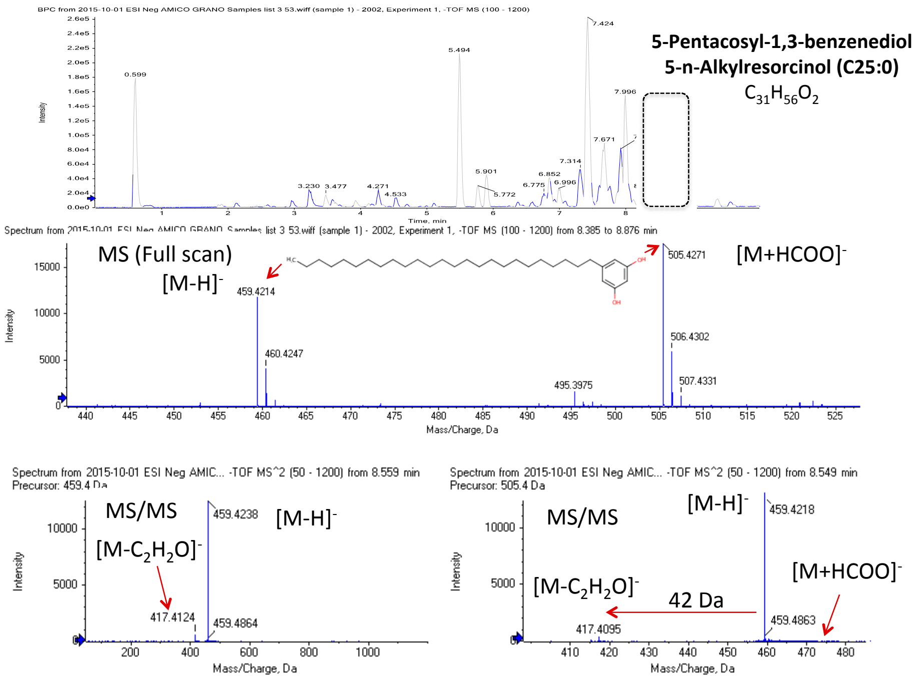

Figure S2. 5-Pentacosyl-1,3-benzenediol 5-n-Alkylresorcinol (C25:0) identification workflow; (i) Base Peak Chromatogram; (ii) MS spectra from 8.38 to 8.87 and (iii) *m*/*z* 459.4214 (deprotonated) and *m*/*z* 505.4271 ($[M + HCOO]^-$) MS/MS pathways.
